# Colorectal Cancer Survival in German–Danish Border Regions—A Registry-Based Cohort Study

**DOI:** 10.3390/cancers15184474

**Published:** 2023-09-08

**Authors:** Christiane Rudolph, Gerda Engholm, Ron Pritzkuleit, Hans H. Storm, Alexander Katalinic

**Affiliations:** 1 Institute for Cancer Epidemiology, University of Lübeck, Ratzeburger Allee 160, 23562 Lübeck, Germany; 2 Danish Cancer Society, Strandboulevarden 49, 2100 København, Denmark

**Keywords:** colorectal neoplasm, radiotherapy, chemotherapy, surgery, neoplasm staging, Kaplan–Meier estimate, proportional hazard models, health care quality, benchmarking

## Abstract

**Simple Summary:**

This study reports updates on colon and rectum cancer survival differences in the German–Danish border region and investigates the effects of tumor stage at diagnosis and first-line treatment on cancer survival. Based on cancer registry data from 2004 to 2016, we estimated the risk of dying from all causes after a colon or rectum cancer diagnosis while also controlling for stage and treatment. We observed that colon and rectum cancer survival improved in the entire German–Danish border region, but much more in the Danish regions. At first, colon and rectal cancer survival was higher on the German side of the border, but the Danish regions caught up. In the end, colon cancer survival was similar across the border and rectal cancer survival was better in Denmark. Stage and treatment could not explain the observed survival differences. Health care reforms and early detection strategies may also contribute to the regional differences.

**Abstract:**

The aim of this study was (i) to update the reporting of colorectal cancer survival differences over time in the German–Danish border region (Schleswig-Holstein, Southern Denmark, and Zealand) and (ii) to assess the extent to which it can be explained by stage and primary treatment. Incident invasive colorectal cancer cases diagnosed from 2004 to 2016 with a follow-up of vital status through 31 December 2017 were extracted from cancer registries. Analyses were conducted by anatomical subsite and for four consecutive periods. Kaplan–Meier curves and log-rank tests were computed. Cox regression models using data from Schleswig-Holstein from 2004 to 2007 as the reference category were run while controlling for age, sex, stage, and treatment. The cox regression models showed decreasing hazard ratios of death for all three regions over time for both anatomical subsites. The improvement was stronger in the Danish regions, and adjustment for age, sex, stage, and treatment attenuated the results only slightly. In 2014–2016, colon cancer survival was similar across regions, while rectal cancer survival was significantly superior in the Danish regions. Regional survival differences can only partially be explained by differing stage distribution and treatment and may be linked additionally to healthcare system reforms and screening efforts.

## 1. Introduction

Colorectal cancer is the third most common cancer and the second most common cause of cancer death among men and women worldwide [1]. In Europe, diverse incidence and mortality trends were observed and linked to the varying implementation of screening programs [2], while survival has generally tended to improve [3]. Denmark was known for a lower colorectal cancer survival compared to the other Nordic countries and its neighboring country Germany, but remarkable improvements on the national level have been observed [3,4,5,6].

International population-based studies with data from cancer registries such as EUROCARE [7] and CONCORD [8] compared cancer survival across countries and continents for the past decades [3,9,10,11]. The latest CONCORD-3 study included German and Danish cancer registry data and reported survival differences between the countries for colon cancer up until 2014 with a significantly higher relative survival in Germany. Rectal cancer relative survival was reported to be higher in Germany up until 2009. As of 2010–2014, a significantly higher relative survival was reported for rectal cancer in Denmark when compared to Germany. However, the study results are limited by the lack of information about tumor stage as TNM and treatment as major prognostic factors for cancer survival [3]. Within the CONCORD protocol, a high-resolution study on colorectal cancer survival was conducted and clinical data about Dukes’ staging and treatment were successfully collected from seven US registries and nine European countries, but Danish and German data were not included [12].

Meaningful cancer survival comparisons based on German and Danish cancer registry data including tumor stage and treatment are, however, feasible as shown for colorectal cancer [13] and breast cancer [14] with data from 2004–2010 and 2004–2013, respectively. Both countries feature universal health care systems, but these differ substantially in terms of access to specialized care (gatekeeper function of Danish general practitioners versus free choice of physicians in Germany), the implementation of early detection programs, and treatment capacities [15]. It is crucial to take tumor stage (as indicator for early detection) and treatment into account when comparing cancer survival across different health care systems. 

The cross-border collaboration in cancer research and care between the German federal state Schleswig-Holstein and the two bordering Danish regions, Southern Denmark and Zealand, was already established in 2007 [16] and has expanded since. The present study aims (i) to update the reporting of colorectal cancer survival differences over time in the German–Danish border region and (ii) to assess the extent to which they can be explained by tumor stage at diagnosis and type of primary treatment.

## 2. Materials and Methods

Incident invasive colorectal cancer cases (International Classification of Diseases (ICD)-10: C18-C20) from 2004 to 2016 with follow-up of vital status through 31 December 2017 were extracted from the population-based Schleswig-Holstein Cancer Registry and for the Danish regions of Southern Denmark and Zealand from the files created for the Nordic cancer statistics database NORDCAN [17] prior to de-identification. The Danish NORDCAN-data were supplemented with treatment information from the Danish Inpatient Hospital Register and death notifications were obtained from the National Population Register. The Danish databases used record linkage via unique personal identification numbers assigned to every resident in Denmark. In the Schleswig-Holstein cancer registry, probabilistic record linkage [18] is used for linking both death notifications from the residents’ registration office and original death certificates to the cancer registry. Data were coded according to the ENCR-JRC 2015 data call protocol [19], and IACR/IARC-rules were applied in handling multiple primaries [20]. The data sets were merged, and a common database for analysis was constructed. The joint database contained information on diagnosis (ICD-10), month and year of birth, incidence, last known vital status, number of days of survival from incidence to death or end of follow-up by end of 2017 or last known vital status (death, immigration, or end of follow-up), sex, age at diagnosis, basis of diagnosis, topography, morphology, behavior, incidental finding of cancer at autopsy, TNM classification, TNM stage grouping, and primary treatment (surgery, radiotherapy, and systemic therapy (including chemotherapy, targeted therapy, immunotherapy, and hormone therapy) recorded as yes/no/unknown). The TNM classification and staging are based on both clinical and pathological information. In the Schleswig-Holstein Cancer Registry, pTNM is used whenever it is available, while cTNM is used only if pTNM is incomplete or not reported. In Denmark, the highest T, N, and M from either clinical or pathological TNM are chosen from the hospital records within the first 4 months after diagnosis. Information on the year of diagnosis was grouped into four periods (2004–2007, 2008–2010, 2011–2013, 2014–2016) to identify time trends in survival and increase the statistical power of regional estimates. All treatment variables were dichotomized, with categories “no” and “unknown” being pooled into one category. The German treatment variables contain information on the first-line treatments administered to patients, which is usually completed six to nine months after diagnosis. The Danish treatment variables contain treatment administered until six months after diagnosis as registered in the Danish Inpatient Hospital Register. 

Cases notified by death certificates only (DCO), age at diagnosis of 90+ years, and incidental findings of cancer at autopsy were excluded from the analyses. DCO-cases and incidental findings of cancers at autopsy were excluded from survival analysis because a reliable incidence date and thus the length of survival was unknown for these cases. Moreover, stage and treatment were unknown for DCO-cases and incidental findings at autopsy. Individuals aged 90 years or older at diagnosis were excluded from analysis because it was expected that diagnostic and treatment rates were lower in this age group. Analyses were conducted for the anatomical subsites colon (ICD-10: C18) and rectum (IDC-10: C19-20) and for four consecutive periods (2004–2007, 2008–2010, 2011–2013, 2014–2016) separately. Frequencies and proportions of the main characteristics of the study population were used for descriptive analyses. Survival time was defined as the time interval between the date of incidence as the entry date and the date of death as the exit date. Patients with vital status alive at the end of follow-up on 31 December 2017 were censored at that date. The outcome of interest was all-cause mortality since diagnosis. The overall survival of colon and rectal cancer patients in the three project regions was calculated using the Kaplan–Meier method, and curves were plotted accordingly. Log-rank tests were conducted to test for significant survival differences between the regions. The regions were compared pairwise. Whether the underlying assumption of proportional hazards for Cox regression was met was assessed through visual inspection of the Kaplan–Meier curves. Multivariate Cox regression models using data from Schleswig-Holstein from 2004 to 2007 as the reference category were applied to calculate hazard ratios of death from all causes while controlling for age, sex, stage, and treatment for each region and period. Missing values in covariates were included as a separate category in all analyses and entered into the models. Results were considered statistically significant when *p* < 0.05. Analyses were conducted using IBM SPSS Statistics 22. 

## 3. Results

In total, 35,279 colon cancer cases (Schleswig-Holstein (SH): *n* = 20,086; Southern Denmark (SD): *n* = 8638; Zealand (ZL): *n* = 6555) and 18,280 rectal cancer cases (SH: *n* = 10,455; SD: *n* = 4480; ZL: *n* = 3345) were reported to the cancer registries between 2004 and 2016. After exclusion of DCO-cases, incidental findings at autopsy, and patients aged 90 years and older at diagnosis, 32,067 colon (SH: *n* = 17,453; SD: *n* = 8290; ZL: *n* = 6324) and 17,172 rectal cancer cases (SH: *n* = 9554; SD: *n* = 4342; ZL: *n* = 3276) were included in the survival analysis. The proportion of DCO-cases was higher in Schleswig-Holstein (colon: 10.4%; rectum: 6.7%) than in the Danish regions (colon: 1.0%; rectum: 0.4%) (Table 1).

### 3.1. Stage Distribution

**Figure 1:**
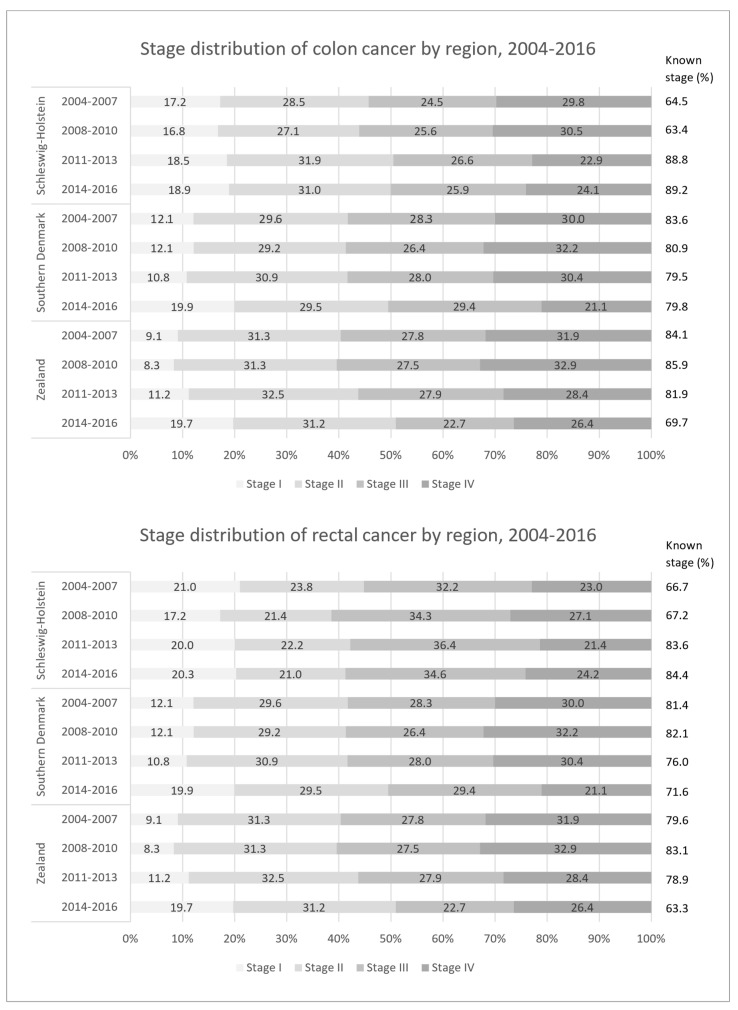
Stage distribution of the included colon and rectal cancer patients for the regions of Schleswig-Holstein, Southern Denmark, and Zealand in 2004–2016.

The stage distribution appeared to be more favorable in Schleswig-Holstein for both colon and rectum cancer. However, there was a shift toward earlier stages in the Danish regions in the latest observation period, 2014–2016. The majority of colorectal cancer cases with known stage were diagnosed in advanced stages III–IV in all three regions. In Schleswig-Holstein, the proportion with available stage information increased over time, while the inverse trend was observed in both Danish regions (Figure 1).

### 3.2. Treatment Patterns

In all regions, surgery was the most frequently performed therapy among colon cancer patients, while radiotherapy was fairly uncommon. The proportion of colon cancer patients receiving systemic therapy increased until 2013 and decreased thereafter. More colon cancer patients received systemic therapy in Southern Denmark (35.2%) and Zealand (43.3%) than in Schleswig-Holstein (25.2%). Among rectal cancer patients, surgery was also the most commonly performed therapy in all regions. Radiotherapy was given to roughly a quarter to one third of the rectal cancer patients. The administration of radiotherapy seemed to decline in the Danish regions, while the proportion of patients receiving systemic therapy increased. In Schleswig-Holstein, the proportion of rectal cancer patients receiving radiotherapy or systemic therapy remained constant (Table 2).

**Figure 2:**
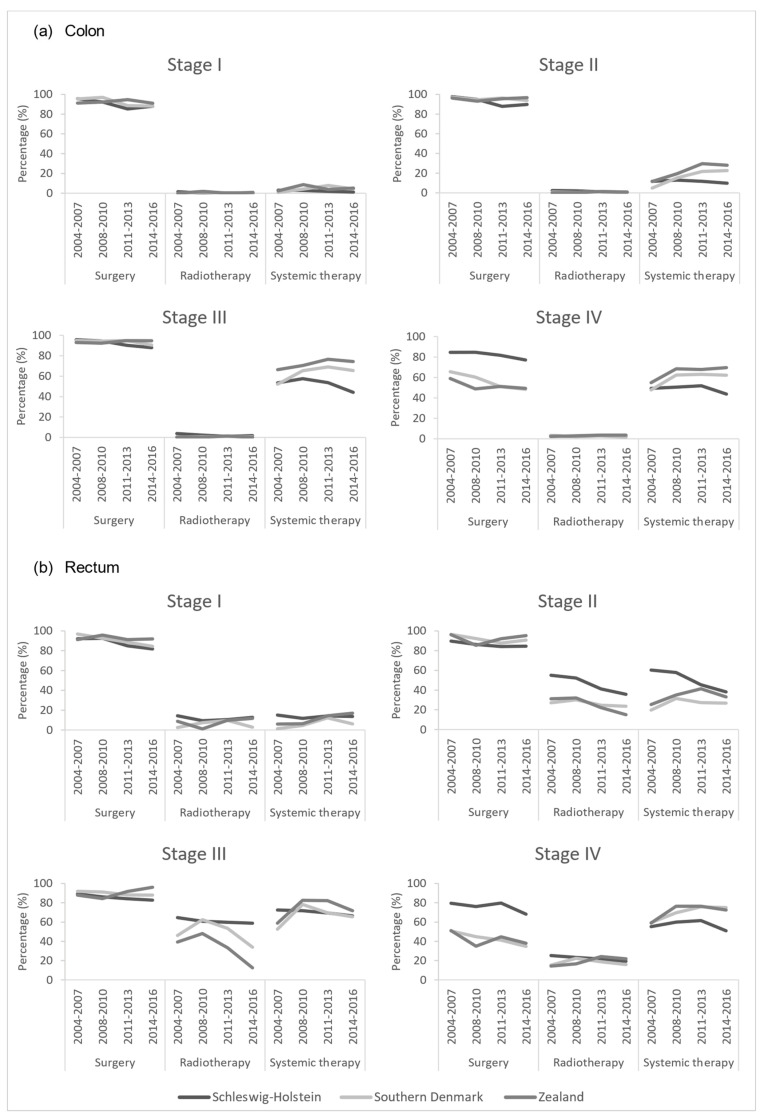
Cancer treatment stratified by stage for colon and rectal cancer in the project regions, 2004–2016.

The stratification of administered treatment by stage showed that treatment for stage I colon cancer was similar in all regions. Among stage II and stage III colon cancers, an increased use of systemic therapies in the Danish regions was observed, while it decreased in Schleswig-Holstein. The treatment choices for stage IV colon cancers varied largely between regions. In Schleswig-Holstein, surgery was the most frequently performed procedure and less common in the Danish regions, while systemic therapies were frequently applied in the Danish region and were less common in Schleswig-Holstein. For rectal cancers, treatment choices were similar across regions for stage I cancers, but differences emerged with advancing stages. Radiotherapy was performed more frequently in Schleswig-Holstein than in the Danish regions among stage II and stage III rectal cancers. Among stage IV rectal cancers, surgery was more often performed in Schleswig-Holstein than in the Danish regions, while systemic therapies were administered more frequently in the Danish regions (Figure 2).

### 3.3. Survival Differences

**Figure 3:**
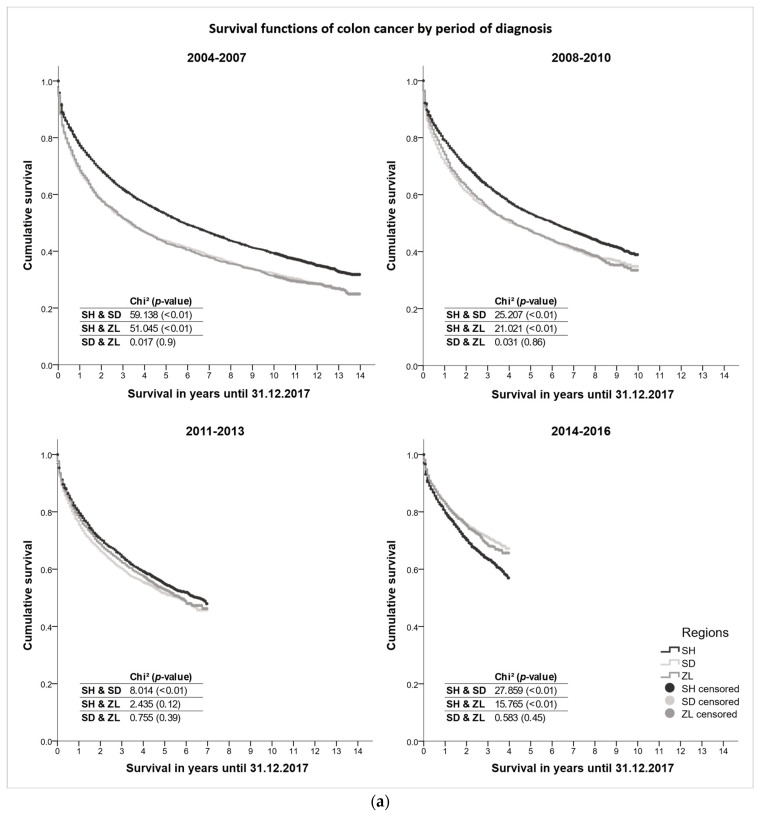

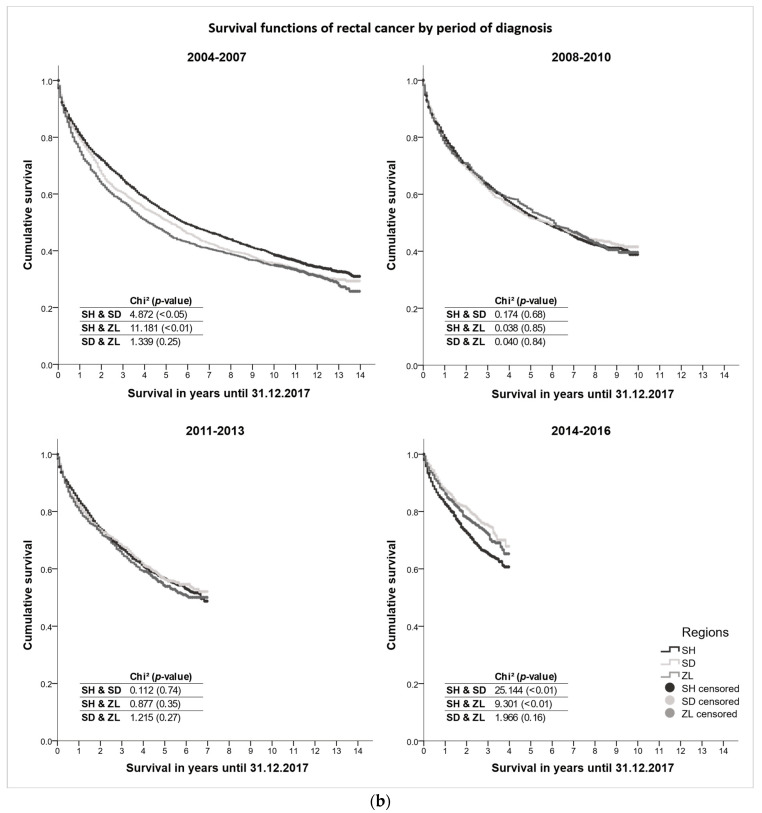
(**a**) Kaplan–Meier curves depicting the colon cancer survival in Schleswig-Holstein, Southern Denmark, and Zealand stratified by period of diagnosis. (**b**) Kaplan–Meier curves depicting the rectal cancer survival in Schleswig-Holstein, Southern Denmark, and Zealand stratified by period of diagnosis.

The Kaplan–Meier survival functions and log rank tests showed, for both colon (Figure 3a) and rectal cancer (Figure 3b), a significantly higher absolute overall survival in Schleswig-Holstein at the beginning of the observation period (2004–2007, and 2008–2010 also for colon) compared to the Danish regions, diminishing survival differences between the three regions in the middle of the observation period, and significantly higher absolute overall survival in the Danish regions in the most recent period (2014–2016). Absolute overall survival improved rapidly in Southern Denmark and Zealand, but small improvements were also observed in Schleswig-Holstein. For colon cancer, the 1/5/10-year absolute survival probability improved in Southern Denmark from 0.68/0.44/0.32 for those diagnosed in 2004-07 to 0.83 (2014-16)/0.52 (2011-13)/0.35 (2008-10) and in Zealand from 0.69/0.43/0.31 in 2004-07 to 0.83 (2014-16)/0.53 (2011-13)/0.33 (2008-10), while it slowly increased in Schleswig-Holstein from 0.77/0.53/0.39 in 2004-07 to 0.80 (2014-16)/0.55 (2011-13)/0.39 (2008-10), respectively. For rectal cancer, very similar trends were observed. The 1/5/10-year absolute rectal cancer survival improved from 0.79/0.51/0.35 among cases diagnosed in 2004-07 to 0.88 (2014-16)/0.57 (2011-13)/0.42 (2008-10) in Southern Denmark and from 0.75/0.46/0.35 in 2004-07 to 0.80 (2014-16)/0.54 (2011-13)/0.40 (2008-10) in Zealand, while Schleswig-Holstein reported increases from 0.81/0.54/0.39 in 2004-07 to 0.83 (2014-16)/0.57 (2011-13)/0.40 (2008-10), respectively (Supplementary Table S1).

**Figure 4:**
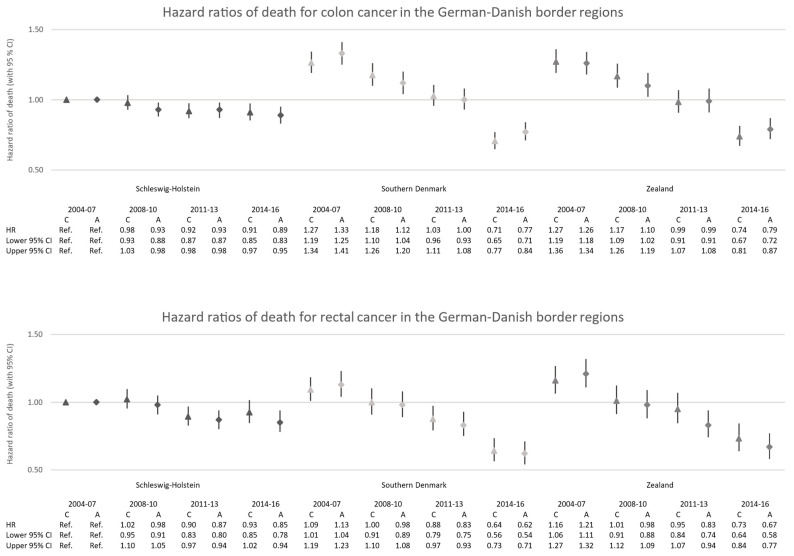
Crude (C) and fully adjusted (A) hazard ratios of death for colon cancer and rectal cancer in Schleswig-Holstein, Southern Denmark, and Zealand compared to cases diagnosed in 2004–2007 in Schleswig-Holstein.

The cox regression models using Schleswig-Holstein’s colon and rectal cancer cases from 2004 to 2007 as the reference category showed decreasing hazard ratios (HRs) of death for all three regions over time for both anatomical subsites (Figure 4).

For cancer patients from Schleswig-Holstein, the crude HR of death decreased to 0.91 (95% CI: 0.85; 0.97) for colon cancer and 0.93 (95% CI: 0.85; 1.02) for rectal cancer in 2014–2016 when compared to 2004–2007. The positive trend is much more prominent in the Danish regions. Though cancer patients residing in Southern Denmark experienced significantly higher crude HRs of death for colon cancer (HR: 1.27, 95% CI: 1.19; 1.34) and rectal cancer (HR: 1.09, 95% CI: 1.01; 1.19) after diagnosis in 2004–2007 when compared to Schleswig-Holstein in the same period, a decreasing trend was observed over the observation time, resulting in significantly lower crude HRs of death for colon cancer (HR: 0.71, 95% CI: 0.65; 0.77) and rectal cancer (HR: 0.64, 95% CI: 0.56; 0.73) in 2014–2016. Similar observations were made for Zealand, where the HR of death started significantly higher for both colon cancer (HR: 1.27, 95% CI: 1.19; 1.36) and rectal cancer (HR: 1.16, 95% CI: 1.06; 1.27) in 2004–2007 in comparison to Schleswig-Holstein and resulted in a significantly lower HR of death for colon cancer (HR: 0.74, 95% CI: 0.67; 0.81) and rectal cancer (HR: 0.73, 95% CI: 0.64; 0.84) in 2014-16.

After adjustment for age at diagnosis, sex, stage, surgery, radiotherapy, and systemic therapy, the observed regional differences between Schleswig-Holstein and the two Danish regions Southern Denmark and Zealand changed only slightly. The HR of death for colon cancer and rectal cancer patients in Southern Denmark and Zealand remained significantly higher after complete adjustment at the beginning of the observation period of 2004-07. In the latest observation period of 2014-16, all three regions showed a significantly lower fully adjusted HR of death for colon cancer (Schleswig-Holstein: HR: 0.89, 95% CI: 0.83; 0.95, Southern Denmark: HR: 0.77, 95% CI: 0.71; 0.84, Zealand: HR: 0.79, 95% CI: 0.72; 0.87) and rectal cancer (Schleswig-Holstein: HR: 0.85, 95% CI: 0.78; 0.94, Southern Denmark: HR: 0.62, 95% CI: 0.54; 0.71, Zealand: HR:0.67, 95% CI: 0.58; 0.77) when compared to Schleswig-Holstein in 2004-07. The regional comparison showed overlapping confidence intervals of the fully adjusted HR for colon cancer among the three regions in the most recent observation period, which indicated no significant survival differences after taking age, sex, stage, and treatment into account, while significantly better HRs were still reported for rectal cancer in the Danish regions after complete adjustment.

## 4. Discussion

This study is, to our knowledge, the first to examine colorectal cancer survival using additional clinical treatment information on the use of surgery, radiotherapy, and systemic therapy in the entire German–Danish border region including for the first time the Danish region of Southern Denmark.

We observed a more favorable stage distribution in Schleswig-Holstein than in Denmark for both cancer sites in 2004–2007, but the proportion of early-stage (TNM stage I + II) colorectal cancer cases increased in the Danish regions in the latest observation period, showing a similar stage distribution to Schleswig-Holstein (Figure 1). The treatment of early-stage colorectal cancer patients was similar across the regions. In the Danish regions, we observed an increase in the use of systemic therapies among colon cancer patients in stages II–IV, exceeding the proportions in Schleswig-Holstein. However, among Danish rectal cancer patients, radiotherapy appears to be less common for stage I–III. Regarding the treatment of stage IV colorectal cancer patients, considerable cross-border differences became apparent. Surgery was more commonly performed among advanced colorectal cancer patients in Schleswig-Holstein, while in the Danish regions, systemic therapies were given more commonly (Figure 2). Multivariate cox regression models showed decreasing hazard ratios of death for all regions in all periods for both colon and rectal. However, survival differences were observed. At the beginning of the observation period (2004–2007), a higher survival was observed in Schleswig-Holstein, but over the course of the observation period, the Danish regions overtook and, in the most recent period (2014–2016), the survival was higher in the Danish regions (Figure 3a,b). Age at diagnosis, sex, stage, surgery, radiotherapy, and systemic therapy could only explain the regional survival differences partially (Figure 4).

The continued positive trend of improving overall colorectal cancer survival in all German–Danish border regions is in line with previous studies. Improving (relative) survival and shrinking survival differences were reported for Germany and Denmark on the national level [3]. The magnitude of improvement was also indicated on the national level, showing steep increases in colorectal cancer survival in Denmark [3,4,5,6], while it stagnated in Germany [3]. A study by Storm et al. examined the relative survival on the regional level for Schleswig-Holstein and the Danish region of Zealand for the periods of 2004–2006 and 2007–2009 and reported superior relative colorectal cancer survival for Schleswig-Holstein, improvements in both regions, and diminishing survival differences especially for rectal cancer [13]. Our findings corroborated these trends on the regional level and seamlessly continued the positive development. The results confirmed the now-statistically significant, better rectal cancer survival after adjustment for various prognostic factors on the Danish side of the border and showed no significant survival differences among colon cancer patients in the German–Danish border region anymore.

The present study covers an observation period of more than a decade, during which scientific advances drove major innovation in clinical oncology and led to changes in health care policies. The exact causes for regional differences and trends in survival cannot be deduced from the present study data, but there are several possible explanations for the observed differences and trends, which may be linked to secondary prevention (early detection), novel therapies, and health care system reforms.

### 4.1. Secondary Prevention for Early Detection

Colorectal cancer screening is a proven public health strategy for early detection, which effectively reduces mortality [21]. A variety of screening tools is available covering stool-based tests (e.g., guaiac-based or immunochemical fecal occult blood test (gFOBT or iFOBT/FIT)) and visual inspection (e.g., computed tomography colonography (CTC), colonoscopy) [22]. While the gFOBT has nowadays been widely replaced by the improved iFOBT/FIT because of its higher sensitivity, visual inspection via colonoscopy remains the most effective tool for reducing colorectal cancer mortality [21,22].

In Germany, an opportunistic colonoscopy screening was introduced in 2002 and, ever since, mortality reductions of 2.6% for men and 3.1% for women were observed [2]. It probably had an impact on the slight but steady improvement in colorectal cancer survival on the German side of the border and might also explain the stage differences observed in 2004 to 2007 between Germany and Denmark. Denmark, however, caught up in terms of early detection and implemented an organized colorectal cancer screening based on the fecal immunochemical test (FIT) in 2014, which led to a mortality reduction in older men with a colorectal cancer diagnosis [23]. We also observed a stage shift toward earlier stages in the latest observation period in 2014 to 2016 in the Danish regions and no relevant remaining stage differences in comparison to Schleswig-Holstein, which may be attributed to the FIT-screening.

### 4.2. Therapeutic Advances

Surgical resection of the malignant tumor and, in advanced stages, the combination with chemotherapy are the cornerstones of colorectal cancer treatment [24]. Over the past decades, existing chemotherapy regimens were improved by adding new agents to the scheme [25,26], or completely new treatment strategies such as gene therapy, targeted therapy or immunotherapy were developed [24]. These novel approaches significantly improved the survival time of colorectal cancer patients, especially among those with advanced stages [24,25,26,27].

Germany is known for a short time to patient access after European Medicines Agency (EMA) approval of new oncological drugs [28]. The fast adoption of novel treatment options may be reflected in the moderate survival improvement in Schleswig-Holstein. The significantly lower survival in the past reported for Denmark may be indicative of the delayed uptake of these new therapies and delayed patient access. Nowadays, however, Denmark is among the fastest adopting countries in Europe [29].

In addition to the accelerated uptake of new oncological drugs, the surgical management of colorectal cancer was improved in Denmark. Laparoscopic surgery was introduced in the early 2000s and, subsequently, the 30-day postoperative mortality after elective major surgery for colorectal cancer decreased significantly from 7.3% in 2001–2002 to 2.8% in 2011 [30]. 

### 4.3. Health Care System Reforms in Denmark

The steep improvements observed in the Danish regions most likely can be linked to a variety of health care reforms. So-called “National Cancer Plans” were implemented and regularly expanded since 2000 [31,32,33,34,35]. While the first National Cancer Plan focused on capacity building for more rapid diagnosis and surgical treatment [32], the second cancer plan introduced patient pathways to accelerate diagnosis and treatment start [33]. The cancer patient pathways were implemented in 2007-09 in order to reduce delays in diagnosis and speed up treatment start [31] and defined a maximum waiting time for initial treatment of 28 days upon the date of referral to the hospital for treatment [36], which was proven to speed-up the diagnostic and treatment processes successfully [31,37].

### 4.4. Strengths and Limitations

The results also have to be discussed in the light of strengths and limitations. The large study population retrieved from cancer registries with complete registration encompassing stage and treatment data is a major strength of the present study [38,39,40,41,42,43,44,45]. Moreover, cancer registry data on colorectal cancer, including stage and treatment information from Schleswig-Holstein and the Danish region South, were merged and jointly analyzed for the first time—now encompassing the entire German–Danish border region. A limitation is the relatively high DCO-rate in Schleswig-Holstein, which may introduce a bias toward better survival [46,47] in Schleswig-Holstein. However, excluding short-term survivors (of 30 and 90 days, respectively) for sensitivity analysis did not alter the results, suggesting minimal bias (Supplement Table S2). Additionally, the high proportion of missing stage information is a potential limitation. But since the Cox regression analysis is not presented on tumor stage level and patients with missing stage are included as a separate category, the overall survival estimates are not likely to be affected. Moreover, treatment is unknown for approximately 12% of the patients, which most probably might be caused by underreporting and may lead to incomplete adjustment. As the statistical adjustment for prognostic factors for cancer survival like age, sex, stage, and treatment attenuated the results, but did not explain the observed survival differences completely, residual confounding is likely to occur. Within the scope of this study, it is not possible to determine the extent to which the various factors discussed (secondary prevention, therapeutic advances, and health care system reforms) contributed to the survival trends. More detailed data would be needed. Unfortunately, information on additional prognostic factors such as screening participation rates, agents and doses used for treatment, socio-economic status, or comorbidities was unavailable. Considering these strengths and limitations, we judge the comparisons made and trends observed to be only marginally biased and thus meaningful.

## 5. Conclusions

Our study shows that colorectal cancer survival improved over time in the entire German–Danish border region, but more so in the Danish regions of Southern Denmark and Zealand. The regional survival differences can only partially be explained by differing stage distribution and treatment and may be linked additionally to health care system reforms and screening efforts. We conclude that the survival improvements observed in all three regions are likely to be true.

## Figures and Tables

**Table 1:** Derivation of analytical sample based on registered colon cancer cases (C18) and rectal cancer cases (C19–C20) in Schleswig-Holstein and the Danish regions of Southern Denmark and Zealand, 2004–2016.

	Schleswig-Holstein		Southern Denmark		Zealand	
	Colon	Rectum	Colon	Rectum	Colon	Rectum
	N (%)	N (%)	N (%)	N (%)	N (%)	N (%)
**2004–2007**						
Patients in cancer register	6192 (100.0)	3386 (100.0)	2280 (100.0)	1302 (100.0)	1698 (100.0)	987 (100.0)
Death certificates only (DCO)/incidental finding at autopsy *	537 (8.7)	191 (5.6)	30 (1.3)	9 (0.7)	20 (1.2)	8 (0.8)
Age 90+ at diagnosis *	305 (4.9)	125 (3.7)	75 (3.3)	38 (2.9)	40 (2.4)	19 (1.9)
Included in survival analysis	5475 (88.4)	3116 (92.0)	2178 (95.5)	1255 (96.4)	1639 (96.5)	960 (97.3)
**2008–2010**						
Patients in cancer register	4781 (100.0)	2423 (100.0)	1808 (100.0)	957 (100.0)	1494 (100.0)	751 (100.0)
Death certificates only (DCO)/incidental finding at autopsy *	549 (11.5)	173 (7.1)	12 (0.7)	5 (0.5)	14 (0.9)	0 (0.0)
Age 90+ at diagnosis *	208 (4.4)	59 (2.4)	67 (3.7)	25 (2.6)	34 (2.3)	7 (0.9)
Included in survival analysis	4134 (86.5)	2219 (91.6)	1730 (95.7)	928 (97.0)	1449 (97.0)	744 (99.1)
**2011–2013**						
Patients in cancer register	4532 (100.0)	2335 (100.0)	2025 (100.0)	1065 (100.0)	1486 (100.0)	706 (100.0)
Death certificates only (DCO)/incidental finding at autopsy *	508 (11.2)	171 (7.3)	17 (0.8)	4 (0.4)	14 (0.9)	2 (0.3)
Age 90+ at diagnosis *	234 (5.2)	91 (3.9)	57 (2.8)	28 (2.6)	43 (2.9)	12 (1.7)
Included in survival analysis	3909 (86.3)	2114 (90.5)	1952 (96.4)	1034 (97.1)	1433 (96.4)	692 (98.0)
**2014–2016**						
Patients in cancer register	4581 (100.0)	2311 (100.0)	2525 (100.0)	1156 (100.0)	1877 (100.0)	901 (100.0)
Death certificates only (DCO)/incidental finding at autopsy *	494 (10.8)	161 (7.0)	23 (0.9)	1 (0.1)	23 (1.2)	2 (0.2)
Age 90+ at diagnosis *	289 (6.3)	78 (3.4)	76 (3.0)	30 (2.6)	55 (2.9)	19 (2.1)
Included in survival analysis	3935 (85.9)	2105 (91.1)	2430 (96.2)	1125 (97.3)	1803 (96.1)	880 (97.7)
**2004–2016**						
Patients in cancer register	20,086 (100.0)	10,455 (100.0)	8638 (100.0)	4480 (100.0)	6555 (100.0)	3345 (100.0)
Death certificates only (DCO)/incidental finding at autopsy *	2088 (10.4)	696 (6.7)	82 (0.9)	19 (0.4)	71 (1.1)	12 (0.4)
Age 90+ at diagnosis *	1036 (5.2)	353 (3.4)	275 (3.2)	121 (2.7)	172 (2.6)	57 (1.7)
Included in survival analysis	17,453 (86.9)	9554 (91.4)	8290 (96.0)	4342 (96.9)	6324 (96.5)	3276 (97.9)

* Sources: Includes also 90+ DCO cases.

**Table 2:** Recorded treatment for colon cancer and rectal cancer for included patients in Schleswig-Holstein, Southern Denmark, and Zealand, 2004–2016.

	Schleswig-Holstein		Southern Denmark		Zealand	
	Colon	Rectum	Colon	Rectum	Colon	Rectum
	N (%)	N (%)	N (%)	N (%)	N (%)	N (%)
**2004–2007**						
Surgery	4571 (83.5)	2417 (77.6)	1835 (84.3)	995 (79.3)	1295 (79.0)	735 (76.6)
Radiotherapy	115 (2.1)	1051 (33.7)	27 (1.2)	340 (27.1)	15 (0.9)	246 (25.6)
Systemic therapy	1330 (24.3)	1294 (41.5)	593 (27.2)	446 (35.5)	571 (34.8)	370 (38.5)
No treatment/Missing	859 (15.7)	560 (18.0)	232 (10.7)	114 (9.1)	219 (13.4)	113 (11.8)
**2008–2010**						
Surgery	3437 (83.1)	1725 (77.7)	1430 (82.7)	725 (78.1)	1099 (75.8)	535 (71.9)
Radiotherapy	62 (1.5)	730 (32.9)	22 (1.3)	313 (33.7)	22 (1.5)	207 (27.8)
Systemic therapy	1073 (26.0)	983 (44.3)	636 (36.8)	464 (50.0)	657 (45.3)	391 (52.6)
No treatment/Missing	649 (15.7)	357 (16.1)	173 (10.0)	66 (7.1)	168 (11.6)	59 (7.9)
**2011–2013**						
Surgery	3166 (81.0)	1607 (76.0)	1574 (80.6)	795 (76.9)	1142 (79.7)	517 (74.7)
Radiotherapy	49 (1.3)	697 (33.0)	30 (1.5)	335 (32.4)	24 (1.7)	165 (23.8)
Systemic therapy	1081 (27.7)	952 (45.0)	791 (40.5)	512 (49.5)	679 (47.4)	392 (56.6)
No treatment/Missing	667 (17.1)	400 (18.9)	191 (9.8)	75 (7.3)	138 (9.6)	51 (7.4)
**2014–2016**						
Surgery	3137 (79.7)	1496 (71.1)	1968 (81.0)	852 (75.7)	1488 (82.5)	671 (76.3)
Radiotherapy	49 (1.2)	680 (32.3)	27 (1.1)	270 (24.0)	27 (1.5)	177 (20.1)
Systemic therapy	916 (23.3)	864 (41.0)	902 (37.1)	487 (43.3)	771 (42.8)	425 (48.3)
No treatment/Missing	692 (17.6)	424 (20.1)	253 (10.4)	94 (8.4)	155 (8.6)	71 (8.1)
**2004–2016**						
Surgery	14,311 (82.0)	7245 (75.8)	6807 (82.1)	3367 (77.5)	5024 (79.4)	2458 (75.0)
Radiotherapy	275 (1.6)	3158 (33.1)	106 (1.3)	1258 (29.0)	88 (1.4)	795 (24.3)
Systemic therapy	4400 (25.2)	4093 (42.8)	2922 (35.2)	1909 (44.0)	2678 (42.3)	1578 (48.2)
No treatment/Missing	2867 (16.4)	1741 (18.2)	849 (10.2)	349 (8.0)	680 (10.8)	294 (9.0)

## Data Availability

The data presented in this study are available on request from the corresponding author. The data are not publicly available, due to privacy restrictions.

## Supplementary Materials

The following supporting information can be downloaded at https://www.mdpi.com/article/10.3390/cancers15184474/s1, Table S1: Number of patients at risk (NaR) and absolute survival (AS) after 1, 5, and 10 years in Schleswig-Holstein, Southern Denmark, and Zealand stratified by period of diagnosis; Table S2: Cox regression models of hazard ratio (HR) of death for cancer registry data of the German-Danish border regions excluding short-term survivors (crude and adjusted for various individual and prognostic factors).
