# Supplementary material for: Colorectal Cancer Survival in German–Danish Border Regions—A Registry-Based Cohort Study
Source: Cancers (Basel). 2023 Sep 8;15(18):4474. doi: 10.3390/cancers15184474 (PMC10526529; doi:10.3390/cancers15184474)
Supplement: Supplementary file 1 [file cancers-15-04474-s001.zip › Table S1_Absolute survival_V3.pdf]

Table S1. Median survival time (mST) in years, number of patients at risk (NaR) and absolute survival (AS) after 1, 5 and 10 years in Schleswig-Holstein, Southern Denmark and Zealand stratified by period of diagnosis.

		Schleswig-Holstein			Southern Denmark			Zealand		
		1 year	5 years	10 years	1 year	5 years	10 years	1 year	5 years	10 years
**Colon**										
2004-07	mST		5.8			3.4			3.4	
	NaR	5460	3098	2209	2178	1021	738	1639	771	552
	AS	0.77	0.53	0.39	0.68	0.44	0.32	0.69	0.43	0.32
2008-10	mST		6.1			4.2			4.3	
	NaR	4100	2312	295	1730	877	105	1449	743	86
	AS	0.79	0.53	0.39	0.71	0.48	0.35	0.74	0.47	0.33
2011-13	mST		6.5			5.6			5.7	
	NaR	3897	1928	-	1952	902	-	1433	692	-
	AS	0.80	0.55	-	0.76	0.52	-	0.78	0.53	-
2014-16	mST		3			3			3	
	NaR	3925	-	-	2430	-	-	1803	-	-
	AS	0.80	-	-	0.83	-	-	0.83	-	-
**Rectum**										
2004-07	mST		5.9			5.2			4.3	
	NaR	3107	1818	1256	1255	694	478	960	491	353
	AS	0.81	0.54	0.39	0.79	0.51	0.35	0.75	0.46	0.35
2008-10	mST		5.7			5.7			6.2	
	NaR	2204	1247	147	928	521	68	744	438	48
	AS	0.80	0.52	0.40	0.79	0.52	0.42	0.78	0.55	0.40
2011-13	mST		6.2			6			6.5	
	NaR	2109	1074	-	1034	540	-	692	344	-
	AS	0.84	0.57	-	0.83	0.57	-	0.81	0.54	-
2014-16	mST		3			3			3	
	NaR	2101	-	-	1125	-	-	880	-	-
	AS	0.83	-	-	0.88	-	-	0.87	-	-
