# Supplementary material for: Colorectal Cancer Survival in German–Danish Border Regions—A Registry-Based Cohort Study
Source: Cancers (Basel). 2023 Sep 8;15(18):4474. doi: 10.3390/cancers15184474 (PMC10526529; doi:10.3390/cancers15184474)
Supplement: Supplementary file 1 [file cancers-15-04474-s001.zip › Table S2_Sensitivity analysis_V2.pdf]

Table S2. Cox regression models of hazard ratio (HR) of death for cancer registry data of the German-Danish border regions excluding short-term survivors (crude and adjusted for various individual and prognostic factors).

		**Excluding short-term survivors <30 days**		**Excluding short-term survivors <90 days**	
		Crude Model	*Fully adjusted*	*Crude Model*	*Fully adjusted*
		HR [95 % CI]	HR [95 % CI]	HR [95 % CI]	HR [95 % CI]
**Colon**	SH 04-07	Ref.	Ref.	Ref.	Ref.
	SD 04-07	1.26 [1.18; 1.34]	1.33 [1.25; 1.42]	1.21 [1.13; 1.30]	1.30 [1.21; 1.39]
	ZL 04-07	1.27 [1.18; 1.36]	1.23 [1.15; 1.32]	1.23 [1.15; 1.33]	1.18 [1.09; 1.27]
	SH 08-10	0.99 [0.93; 1.04]	0.93 [0.88; 0.99]	0.99 [0.93; 1.05]	0.93 [0.88; 0.99]
	SD 08-10	1.19 [1.11; 1.28]	1.12 [1.04; 1.20]	1.15 [1.06; 1.24]	1.05 [0.96; 1.14]
	ZL 08-10	1.19 [1.11; 1.29]	1.09 [1.01; 1.18]	1.18 [1.09; 1.28]	1.04 [0.95; 1.13]
	SH 11-13	0.94 [0.88; 0.996]	0.93 [0.87; 0.99]	0.97 [0.91; 1.03]	0.94 [0.88; 1.01]
	SD 11-13	1.07 [0.99; 1.15]	1.03 [0.91; 1.09]	1.04 [0.96; 1.13]	0.98 [0.90; 1.06]
	ZL 11-13	1.03 [0.94; 1.12]	0.99 [0.92; 1.09]	1.03 [0.94; 1.13]	0.96 [0.87; 1.05]
	SH 14-16	0.93 [0.87; 0.999]	0.90 [0.84; 0.97]	0.95 [0.88; 1.02]	0.92 [0.85; 0.99]
	SD 14-16	0.72 [0.65; 0.79]	0.77 [0.70; 0.85]	0.73 [0.66; 0.81]	0.77 [0.69; 0.85]
	ZL 14-16	0.78 [0.70; 0.86]	0.81 [0.73; 0.89]	0.78 [0.70; 0.87]	0.78 [0.69; 0.87]
**Rectum**	SH 04-07	Ref.	Ref.	Ref.	Ref.
	SD 04-07	1.12 [1.03; 1.22]	1.17 [1.08; 1.27]	1.11 [1.02; 1.21]	1.18 [1.08; 1.28]
	ZL 04-07	1.18 [1.07; 1.29]	1.23 [1.13; 1.35]	1.17 [1.06; 1.28]	1.24 [1.13; 1.36]
	SH 08-10	1.05 [0.97; 1.13]	1.0 [0.93; 1.07]	1.04 [0.96; 1.12]	0.98 [0.91; 1.06]
	SD 08-10	1.02 [0.92; 1.13]	1.0 [0.90; 1.10]	1.01 [0.91; 1.12]	0.99 [0.89; 1.09]
	ZL 08-10	1.05 [0.94; 1.17]	1.0 [0.90; 1.12]	1.02 [0.92; 1.15]	0.97 [0.86; 1.08]
	SH 11-13	0.92 [0.84; 0.99]	0.88 [0.81; 0.95]	0.92 [0.84; 0.999]	0.87 [0.79; 0.95]
	SD 11-13	0.92 [0.82; 1.02]	0.86 [0.78; 0.96]	0.91 [0.81; 1.02]	0.85 [0.76; 0.95]
	ZL 11-13	0.98 [0.87; 1.11]	0.85 [0.75; 0.96]	0.99 [0.87; 1.12]	0.83 [0.73; 0.95]
	SH 14-16	0.95 [0.86; 1.04]	0.87 [0.79; 0.96]	0.96 [0.87; 1.06]	0.88 [0.79; 0.98]
	SD 14-16	0.68 [0.59; 0.77]	0.64 [0.56; 0.74]	0.71 [0.62; 0.82]	0.67 [0.58; 0.78]
	ZL 14-16	0.77 [0.66; 0.89]	0.69 [0.60; 0.80]	0.78 [0.67; 0.92]	0.70 [0.60; 0.82]
